# Comparison of the Anabolic Effects of Reported Osteogenic Compounds on Human Mesenchymal Progenitor-Derived Osteoblasts

**DOI:** 10.3390/bioengineering7010012

**Published:** 2020-01-21

**Authors:** Robert Owen, Hossein Bahmaee, Frederik Claeyssens, Gwendolen C. Reilly

**Affiliations:** 1 Department of Materials Science and Engineering, INSIGNEO Institute for In Silico Medicine, The Pam Liversidge Building, Sir Frederick Mappin Building, Mappin Street, Sheffield S1 3JD, UK; hbahmaee1@sheffield.ac.uk (H.B.); f.claeyssens@sheffield.ac.uk (F.C.); g.reilly@sheffield.ac.uk (G.C.R.); 2 Department of Materials Science and Engineering, University of Sheffield, Kroto Research Institute, Sheffield S3 7HQ, UK

**Keywords:** Matrix mineralisation, osteoblasts, bone formation, mesenchymal stem cells, osteoporosis, bone tissue engineering, menaquinone-4, vitamin K

## Abstract

There is variability in the reported effects of compounds on osteoblasts arising from differences in experimental design and choice of cell type/origin. This makes it difficult to discern a compound’s action outside its original study and compare efficacy between compounds. Here, we investigated five compounds frequently reported as anabolic for osteoblasts (17β-estradiol (oestrogen), icariin, lactoferrin, lithium chloride, and menaquinone-4 (MK-4)) on human mesenchymal progenitors to assess their potential for bone tissue engineering with the aim of identifying a potential alternative to expensive recombinant growth factors such as bone morphogenetic protein 2 (BMP-2). Experiments were performed using the same culture conditions to allow direct comparison. The concentrations of compounds spanned two orders of magnitude to encompass the reported efficacious range and were applied continuously for 22 days. The effects on the proliferation (resazurin reduction and DNA quantification), osteogenic differentiation (alkaline phosphatase (ALP) activity), and mineralised matrix deposition (calcium and collagen quantification) were assessed. Of these compounds, only 10 µM MK-4 stimulated a significant anabolic response with 50% greater calcium deposition. Oestrogen and icariin had no significant effects, with the exception of 1 µM icariin, which increased the metabolic activity on days 8 and 22. 1000 µg/mL of lactoferrin and 10 mM lithium chloride both significantly reduced the mineralised matrix deposition in comparison to the vehicle control, despite the ALP activity being higher in lithium chloride-treated cells at day 15. This demonstrates that MK-4 is the most powerful stimulant of bone formation in hES-MPs of the compounds investigated, highlighting its potential in bone tissue engineering as a method of promoting bone formation, as well as its prospective use as an osteoporosis treatment.

## 1. Introduction

There is a need for new anabolic compounds in the field of bone tissue engineering to enhance the rate of bone formation on scaffolds and to improve tissue regeneration. Under standard osteogenic culture conditions with media supplemented with dexamethasone, β-glycerophosphate, and ascorbic acid [1], it can take months to years for scaffolds to become fully mineralised and bone-like [2]. Although osteogenesis can be enhanced through the use of growth factors such as bone morphogenetic protein 2 (BMP-2), their use is not without limitations. The major adverse side effect of BMP-2 treatment is ectopic bone formation due to leakage from the implant site; therefore, loading tissue engineering scaffolds for clinical treatments with unconstrained BMP-2 is not an ideal solution [3]. Furthermore, the response of human mesenchymal stem cells (MSCs) to BMP-2 is not consistent between donors [4], and the cost of using recombinant proteins is prohibitive to widespread clinical use. 

This need has resulted in numerous compounds being tested for anabolic effects on bone, many of which have been described in the literature as ‘osteogenic’. However, the lack of standardisation between laboratories and experiments has resulted in conflicting information about these effects. For example, in the case of oestrogen, it has been shown to both accelerate [5] and slow down [6] osteoblast proliferation, upregulate alkaline phosphatase activity [6] but suppress osteocalcin production [6,7], and enhance [8], not affect [9], or only augment mineralisation when applied intermittently [5]. This variation makes it difficult to predict the action of each compound outside the original study and to compare the relative efficacy between therapeutics. 

These varied findings arise from numerous factors. Some are unavoidable; for example, it is unreasonable to assume that all laboratories could use identical media formulations. Another source of variability is that separate studies investigate dissimilar concentrations of therapeutics and use different assays and time points to assess outcomes. Whilst this renders direct comparisons unreliable, it still allows for more general comparisons to be made; for example, was the differentiation or matrix production still enhanced? 

The final and perhaps most important source of variability is the use of different cell lines to represent the cell type of interest and donor variability in primary cells. On the proviso that multiple donors are tested within the same study, donor variability is desirable as it represents the wider human population; with single donor studies it cannot be ascertained whether the observed effects are donor-specific or not. Animal-derived cell lines are often used in lieu of human ones in cell culture, but genetic differences mean that observed responses cannot be assumed to also occur in human cells. Additionally, using osteoblasts as an example cell type, osteosarcoma cells such as MG-63 and Saos-2 are often reported as being osteoblasts or osteoblast-like despite the fact that they have abnormal, cancerous growth characteristics and cannot mirror the whole range of osteoblast phenotypic changes. Furthermore, there are inconsistencies in their reported ability to deposit a mineralised extracellular matrix, including some reports stating that a mineralised matrix is not deposited by these cell types [10,11]. Therefore, it is difficult to discern whether the effects reported on these cell lines are transferable to primary osteoblastic cells. Importantly, these sources of variability combine and result in a collection of literature that states that the same compound can promote, inhibit, or have no effect on bone formation. 

In this study, in order to minimise this variation and improve reproducibility, commercially available Cellartis® human embryonic stem cell-derived mesenchymal progenitor cells 002.5 (hES-MPs) were used for all experiments in the precise recommended medium. This cell type was selected for two reasons; first, the commercial availability allows for the experiments to be more reproducible outside the original laboratory in comparison to primary cells; and second, it was selected due to its extensive use as a model osteoblast-lineage cell in tissue engineering studies. This cell line is a homogeneous, robust source of mesenchymal progenitors that has been demonstrated to be well representative of adult-derived mesenchymal stem cells (MSCs) and can be differentiated into osteogenic, chondrogenic, and adipogenic lineages [12,13]. With regards to using it as a model osteoblast-lineage cell, hES-MPs differentiate from mesenchymal progenitors into osteoblasts under osteogenic culture conditions. There is increasing alkaline phosphatase (ALP) activity over time, the deposition of mineralised extracellular matrix, expression of osteocalcin, osteopontin and MSC markers CD146, CD105, and CD90, and overall gene expression levels highly similar to adult MSCs [12,14,15]. Previously, they have been used in numerous bone tissue engineering studies [16,17,18,19,20,21,22,23], the development of bioreactors [24], assessments of the effects of mechanical, chemical, and geometrical differentiation cues [25,26,27,28], and for bone toxicity testing [29] demonstrating their suitability for this study. 

Here, five compounds (17β-estradiol (oestrogen), icariin, lactoferrin, lithium chloride, and menaquinone-4), identified in the literature as having an anabolic effect on osteoblasts, are compared at concentrations over two orders of magnitude encompassing the reported efficacious range. Oestrogen, icariin (a phytoestrogen), and lactoferrin are reported to promote osteoblast proliferation and differentiation. Treatment with lithium chloride is associated with a higher bone mineral density and reduced fracture risk, and menaquinine-4 is a form of vitamin K that is reported to stimulate osteoblast differentiation and that facilitates mineralisation [5,30,31,32,33,34,35,36,37,38,39,40,41,42,43]. An identical dosing regimen and cell culture protocol are used for each compound to allow direct comparisons to be made, with the proliferation, osteogenic differentiation, and mineralised extracellular matrix deposition being assessed. Although these compounds are less commonplace than better known promoters of osteogenesis such as bone morphogenetic protein 2 (BMP-2), any verified anabolic effect would represent a cheaper, more accessible approach to improving bone formation in the field of tissue engineering and a potential therapeutic for conditions such as osteoporosis.

## 2. Materials and Methods 

All materials were sourced from Sigma-Aldrich, UK, unless otherwise stated.

### 2.1. Cell Culture

Cellartis® human embryonic stem cell-derived mesenchymal progenitor cells 002.5 (hES-MPs, Takara-Bio, France) were used for all cell culture experiments (passage 6−7). The cells were passaged in gelatin coated flasks at 37 °C, 5% CO2 and >90% humidity in the recommended expansion media (EM): basal media (BM, Dulbecco’s Modified Eagle Medium (DMEM, Gibco, UK), supplemented with 10% qualified, heat inactivated, US origin foetal bovine serum (FBS, Gibco, UK, cat# 16140), 100 U/mL penicillin, and 100 μg/mL streptomycin (Gibco, UK)), supplemented with 4 ng/mL basic fibroblast growth factor (FGF, Peprotech, UK). 

For all experiments, hES-MPs were seeded in gelatin-coated 24-well plates at 30,000 cells/cm^2^ in basal media and left overnight to attach. Following a metabolic activity assay on day 1, cells were maintained in osteogenesis induction media (OIM: BM supplemented with 50 μg/mL ascorbic acid 2-phosphate (AA2P), 5 mM beta-glycerolphosphate (βGP), and 100 nM dexamethasone) containing the appropriate compound for the remainder of the experiment. Full media changes were performed three times a week, with the metabolic activity determined on days 1, 8, 15, and 22, the alkaline phosphatase activity and total DNA measured on day 15, and the calcium and collagen deposition quantified on day 22. 

### 2.2. Compound Preparation

Five different compounds were compared in this study: lactoferrin, lithium chloride, 17β-estradiol (oestrogen), icariin, and menaquinone-4 (MK-4). Each was applied at three concentrations spanning three orders of magnitude in order to encompass the range reported as anabolic in the literature (Table 1). The vehicle concentration was controlled for all conditions.

To create stock solutions, lactoferrin from bovine colostrum (cat# L4765, ~90 kDa) was dissolved in BM at 5 mg/mL, and lithium chloride (cat# L9650) was dissolved in BM at 1 M. Oestrogen, icariin, and MK-4 have poor solubility in aqueous solutions such as culture media. Therefore, 17β-estradiol (cat# E2758) was dissolved in ethanol (Fisher Scientific, UK), and then diluted in BM to a final concentration of 10 μM 17β-estradiol and 2% ethanol. Icariin and MK-4 were both dissolved in dimethyl sulfoxide (DMSO) at 10 mM. All stock solutions were sterilised by 0.2 μM filtration using ultra-low adsorption polyether sulfone filters (Whatman® Puradisc), divided into single-use aliquots and stored at −20 °C.

### 2.3. Metabolic Activity 

The cell metabolic activity was evaluated by resazurin reduction (RR). Culture media was exchanged for RR working solution (10 vol% RR stock solution (1 mM resazurin sodium salt in deionised water (diH_2_O)) in BM), and the well plate was wrapped in foil and incubated for 4 hours. 200 μL of the reduced solution was transferred in triplicate to a 96-well plate and read on a plate reader (Tecan infinite 200-pro) at λ_ex_: 540 nm and λ_em_: 590 nm. The wells were rinsed with phosphate buffered saline (PBS) before adding fresh medium.

### 2.4. Alkaline Phosphatase Activity and DNA Quantification

The alkaline phosphatase (ALP) activity determination and total DNA quantification were performed on cell lysates on day 15. To digest, the media was removed and the wells were washed twice with PBS. 1 mL of cell digestion buffer (10 vol% cell assay buffer (1.5 M Tris-HCl, 1 Mm ZnCl_2_, 1mM MgCl_2_ in diH_2_O), 1%Triton-X100 in diH_2_O) was added to the wells and refrigerated overnight. The well plates were then freeze-thawed three times (−80 °C/37 °C) before scraping the wells and transferring the contents to microcentrifuge tubes. Lysates were centrifuged at 6700 RCF for 5 minutes before homogenising the supernatant. 

The ALP activity was determined using the Pierce^TM^ PNPP substrate kit (ThermoFisher Scientific, UK) according to the manufacturer’s instructions. Briefly, 20 μL of lysate was combined with 180 μL of substrate (p-nitrophenol phosphate, pNPP) in a 96-well plate. The change in absorbance was measured using a plate reader (Tecan infinite 200-pro) at a wavelength of 405 nm every 90 seconds for 45 min. The ALP activity is expressed as nmol of p-nitrophenol per minute (nmol pNP/min), assuming that one absorbance value equals 19.75 nmol of product. This activity was normalised to the total DNA content per lysate.

DNA was quantified using the Quant-iT^TM^ high sensitivity dsDNA kit (ThermoFisher Scientific, UK), according to manufacturer’s instructions. Briefly, 10 μL of lysate was combined with 90 μL of substrate in a black 96-well plate. The plates were then shaken to aid the DNA-substrate conjugation, left at room temperature for 10 minutes, then shaken again before measuring the fluorescence (𝜆_𝑒𝑥_: 485 nm, 𝜆_𝑒𝑚_: 535 nm). The shaking and fluorescence were performed and measured using a plate reader (Tecan infinite 200-pro). The fluorescence was converted to ng of DNA using a standard curve and was scaled to the total lysate volume. 

### 2.5. Calcium and Collagen Quantification

The wells were fixed by removing the culture media, washing twice with PBS, adding 3.7% formaldehyde for 20 minutes, then washing twice in diH_2_O. The calcium quantification was performed by Alizarin Red S (ARS) staining. ARS was dissolved at 1 w/v% in diH2O, filtered (0.45 µm), and the pH was adjusted to 4.1. The wells were stained for 30 minutes before washing away residual ARS with diH_2_O. The wells were air-dried and photographed before destaining with 1 mL of 5% perchloric acid. 150 µL was then transferred in triplicate to a 96-well plate and read at an absorbance of 405 nm. (Tecan infinite 200-pro). The concentration of ARS was determined via a standard curve.

After calcium staining, the wells were washed again with diH_2_O before staining for collagen with direct red 80 (DR80). DR80 was dissolved in saturated picric acid at 0.1 w/v% and filtered (0.45 µm). The wells were stained for 1 hour before washing away residual DR80 with diH2O. The wells were air-dried and photographed before destaining with 1 mL of 0.2 M sodium hydroxide:methanol in 1:1 ratio. 150 µL was then transferred in triplicate to a 96-well plate and read at an absorbance of 540 nm. (Tecan infinite 200-pro). The concentration of DR80 was determined via a standard curve. 

### 2.6. Statistical Analysis

All statistical analyses were performed in GraphPad Prism (version 7.00). For the resazurin reduction assays, the results were normalised to the mean of the vehicle control at each time point for each repeat. Significant differences were evaluated by a two-way analysis of variance (ANOVA) with Dunnett’s multiple comparisons test comparing each compound concentration to the vehicle control. For all other assays, the results were normalised to the mean of the vehicle control for each repeat. Given that not all of the groups fitted a Gaussian distribution (Shapiro-Wilk normality test), significant differences were evaluated via the Kruskal-Wallis test with Dunn’s multiple comparisons test to compare each compound concentration to the vehicle control. All graphs are presented as the mean ± standard deviation, and significant differences are indicated on the graphs and in the legends. Differences were considered significant when p < 0.05 (*). Other p-values designated in this manuscript are ** = p < 0.01, *** = p < 0.001, **** = p < 0.0001. 

## 3. Results

### 3.1. Establishing the Baseline Response of hES-MPs to Osteogenic Conditions

**Figure 1:**
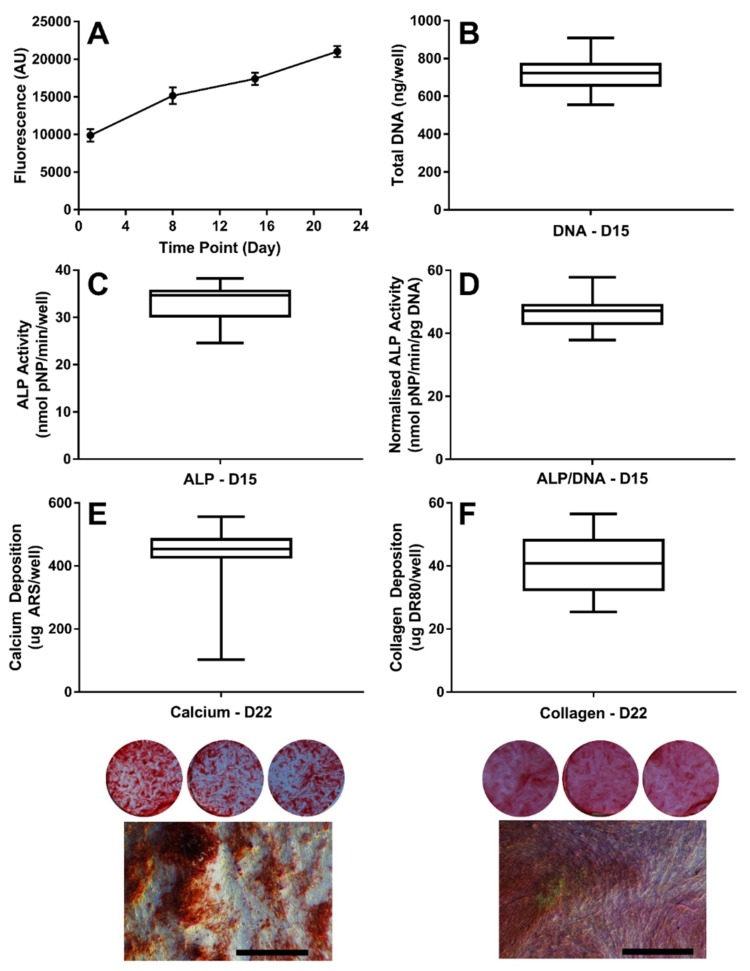
The baseline response of hES-MPs to osteogenesis induction media (OIM) over 22 days. (**A**) The metabolic activity. Boxplots showing minimum, 1st quartile, mean, 3rd quartile and maximum for (**B**) the total DNA, (**C**) ALP activity, (**D**) normalised ALP activity, (**E**) calcium deposition, and (**F**) collagen deposition. (n = 48). Whole well images (diameter ~15 mm) and a high magnification phase contrast image (scale bar: 100 μm) show typical matrix deposition characteristics.

In each experimental plate, hES-MPs were cultured under standard osteogenic conditions (OIM), OIM with the appropriate vehicle control for the compound of interest, and OIM with the three compound concentrations. The data from cells cultured in OIM was collated across all experimental plates to establish a baseline response of hES-MPs to standard osteogenic conditions (Figure 1). As it has been previously demonstrated that hES-MPs do not undergo osteogenic differentiation when maintained in basal media, this was not repeated here [16,18,25,28,44]. As expected, they proliferated to confluence and underwent differentiation into osteoblasts. On day 15, the total DNA per well was 717.9 ± 87.8 ng, and the ALP activity per well was 33.17 ± 3.72 nmol pNP/min, yielding a normalised ALP activity of 46.49 ± 4.82nmol pNP/min/pg DNA. In the third week of culture, hES-MPs began to deposit a mineralised, collagenous extracellular matrix. On day 22, the calcium deposition per well retained 435.8 ± 96.13 μg of ARS, and the collagen deposition per well retained 40.10 ± 8.99 μg of DR80. There was a high concordance between repeats for each assay used to establish a baseline response, demonstrating the reproducibility of the osteogenic differentiation of hES-MPs. Although the calcium deposition had a wide range between the minimum and maximum, only 4 values were below the 10^th^ percentile, and the narrow interquartile range shows the low spread of the data. 

### 3.2. Determination of Appropriate Concentration of the DMSO Vehicle 

**Figure 2:**
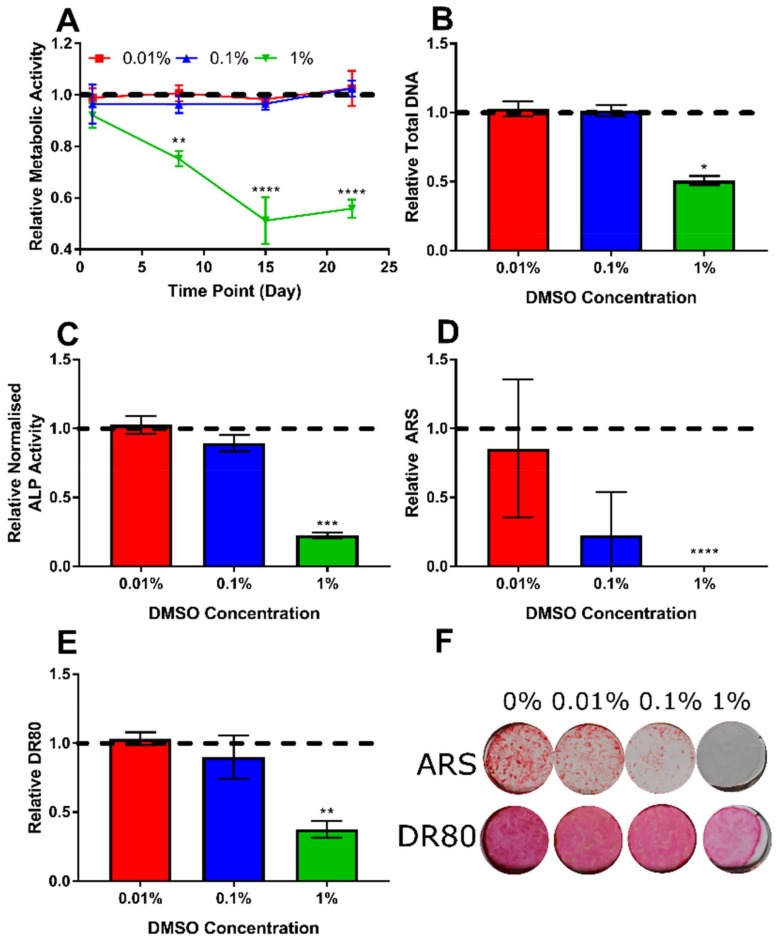
The effect of DMSO normalised to 0% (horizontal dashed line). (**A**) The metabolic activity normalised to 0% at each time point, (**B**) D15 total DNA, (**C**) D15 normalised ALP activity, (**D**) D22 ARS, (**E**) D22 DR80, and (**F**) representative whole well photographs of ARS and DR80 staining at each concentration (diameter ~15 mm). (n = 8). No significant effect on any parameter for 0.01% or 0.1%. At 1% DMSO, the metabolic activity was significantly reduced from D7 onwards, with ALP, DNA, mineral, and collagen also all being significantly lower.

Icariin and MK-4 are not readily soluble in aqueous media; therefore, it was necessary to identify the lowest concentration of DMSO that permitted the application of the highest required dose of the compound. It was identified that only at 1% DMSO were any of the analysis parameters significantly affected (Figure 2); therefore, either 0.1% or 0.01% DMSO would be acceptable vehicle control concentrations. However, a minimum of 0.1% DMSO was required to achieve a 10 µM concentration of MK-4. 17β-estradiol also has a poor aqueous solubility and was therefore first dissolved in ethanol before being diluted in BM. However, due to the very low final concentration of ethanol (0.02%) during the experiments, no effects were detectable (data not shown).

### 3.3. The Highest Concentrations of Lactoferrin and Lithium Chloride SSignificantly Reduce Metabolic Activity

**Figure 3:**
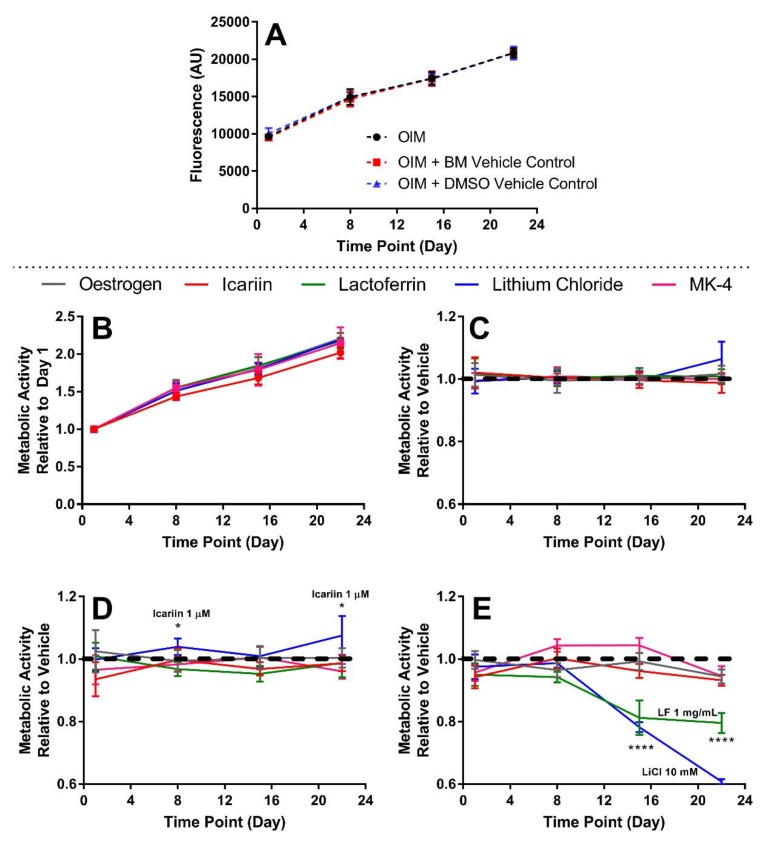
The effect of each compound and concentration on the metabolic activity. No significant difference in the metabolic activity for hES-MPs cultured in (**A**) OIM vs. BM/DMSO vehicle controls (n = 40), and (**B**) the vehicle controls for each compound, normalised to day 1 (n = 8). (C–E) The effect of each compound at the (**C**) lowest, (**D**) middle, and (**E**) highest concentrations on the metabolic activity, normalised to their respective vehicle control at each time point (horizontal dashed line). (n = 8). Lactoferrin (1 mg/mL) and lithium chloride (10 mM) significantly reduced the metabolic activity from day 15 onwards (p < 0.0001). 1 µM icariin significantly increased the metabolic activity on days 8 and 22 (p < 0.05). No other condition significantly deviated from the vehicle control growth curve.

A comparison of the metabolic activities of hES-MPs cultured in OIM versus the vehicle controls was performed initially, finding no significant difference at any time point (Figure 3A). When investigating the effects of compounds, the baseline metabolic activity was determined on day 1 prior to the initial application, then in each subsequent week until the end of the study. There were no differences in growth in the vehicle controls for each compound (Figure 3B). The concentrations of compound that caused a significant deviation from their respective vehicle control growth curve were lactoferrin at 1 mg/mL and lithium chloride at 10 mM (Figure 3E), which caused a significantly lower metabolic activity from day 14 onwards (p < 0.0001), and 1 µM icariin, which induced a significantly higher metabolic activity on days 8 and 22 (p < 0.05, Figure 3D). 

### 3.4. Total DNA Confirms High Concentrations of Lactoferrin and Lithium Chloride Reduce Cell Number

**Figure 4:**
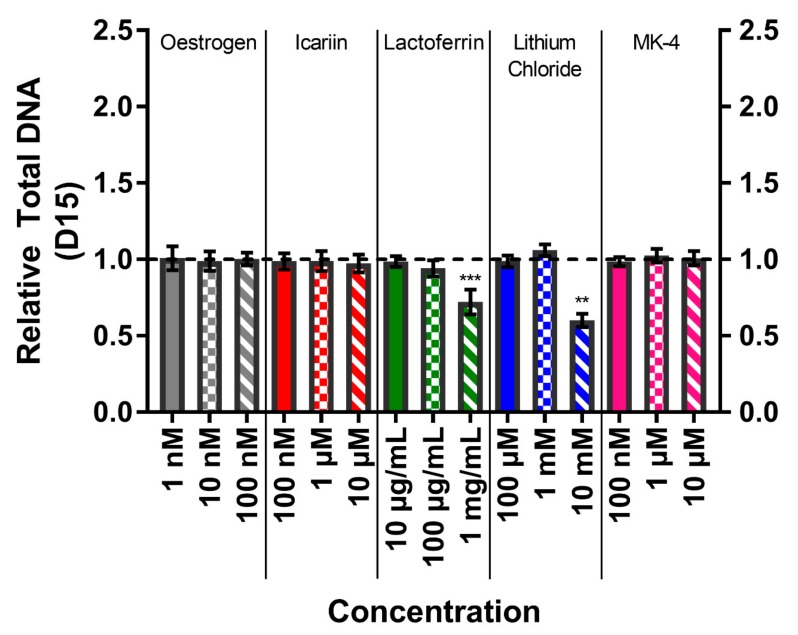
The effect of each compound and concentration on the total DNA per well at day 15, normalised to their respective vehicle control (horizontal dashed line). The treatment with lactoferrin (1 mg/mL) and lithium chloride (10 mM) significantly lowered the total DNA (p < 0.001 & P <0.01, respectively). No other condition significantly deviated from the vehicle control. (n = 8).

The total DNA was quantified on day 15 to compare to metabolic activity and normalise the ALP activity (Figure 4). The total DNA amounts per well for the vehicle controls were 723.0 ± 70.1 ng DNA. The total DNA correlated with the metabolic activity, with treatments of lactoferrin at 1 mg/mL and lithium chloride at 10 mM causing significantly reduced total DNA in comparison to their respective vehicle controls (p < 0.01). 

### 3.5. The Highest Concentrations of Lithium Chloride Significantly Enhance ALP Activity

**Figure 5:**
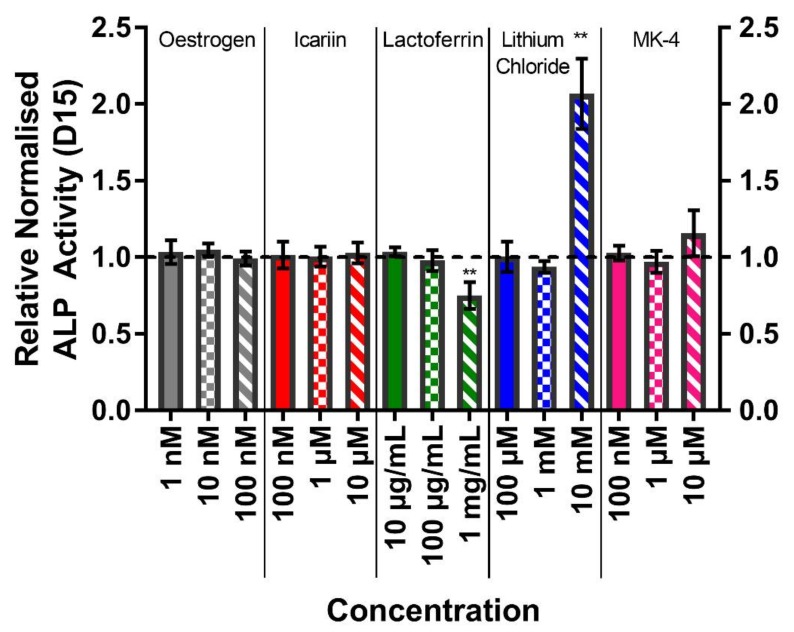
The effect of each compound and concentration on the normalised ALP activity at day 15, normalised to their respective vehicle control (horizontal dashed line). The treatment with lactoferrin (1 mg/mL) resulted in a significantly lower normalised ALP activity, whilst the treatment with lithium chloride (10 mM) elevated the normalised ALP activity by 100% (both p < 0.01). No other condition significantly differed from the vehicle control. (n = 8).

The alkaline phosphatase activity was quantified on day 15 as a marker of osteogenic differentiation (Figure 5). The normalised ALP activities for the vehicle controls were 32.34 ± 3.34 nmol pNP/min/pg DNA. As with markers of cell number, only lactoferrin at 1 mg/mL and lithium chloride at 10 mM significantly differed from the vehicle control. However, whilst the normalised ALP activity was lower after the treatment with lactoferrin (p < 0.01), it was higher (two-fold) with lithium chloride (p < 0.01). In the case of lactoferrin, both the total ALP activity and total DNA were lower than the vehicle control, whereas the treatment with 10 mM lithium chloride resulted in a significantly higher total ALP activity despite the lower total DNA per well, hence the dramatic change (data not shown). 

### 3.6. Menaquinone-4 Significantly Increases Mineral Deposition

**Figure 6:**
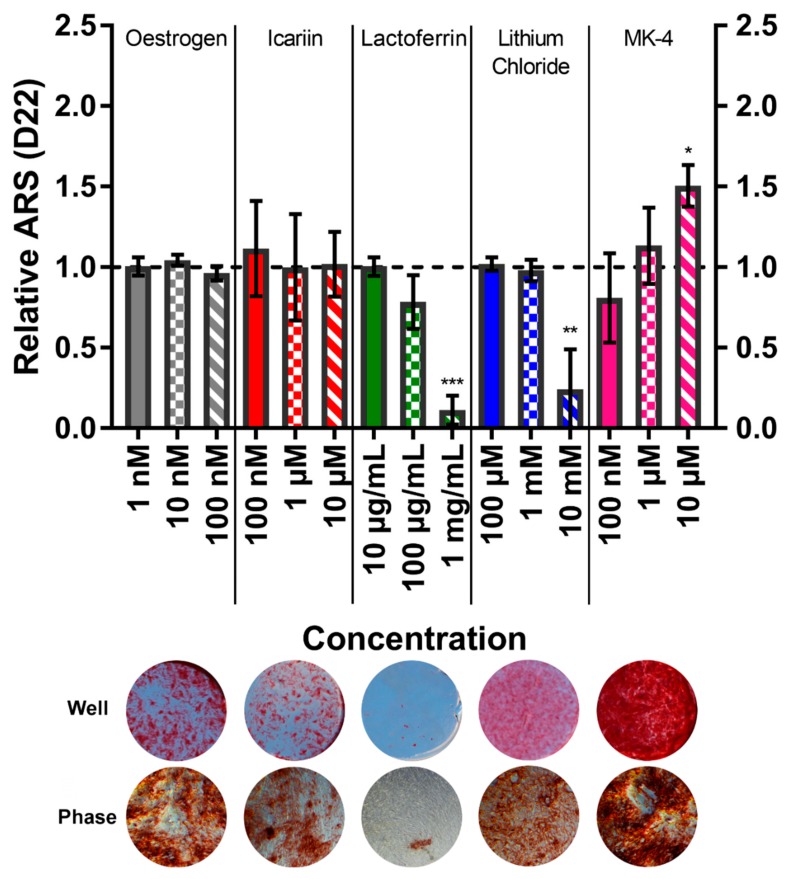
(**Top**) The effect of each compound and concentration on the calcium deposition at day 22, normalised to their respective vehicle control (horizontal dashed line). The treatment with lactoferrin (1 mg/mL) and lithium chloride (10 mM) resulted in a significantly lower calcium deposition in comparison to the vehicle control (p < 0.001 & p < 0.01, respectively), whilst the MK-4 (10 µM) treatment resulted in a 50% greater calcium deposition (both p < 0.05). No other condition significantly deviated from the vehicle control. (n = 8). (**Bottom**) Representative whole well images (diameter ~15 mm) and high magnification (40×) phase contrast images (diameter 200 μm) of ARS staining of each compound at the highest concentration applied.

**Figure 7:**
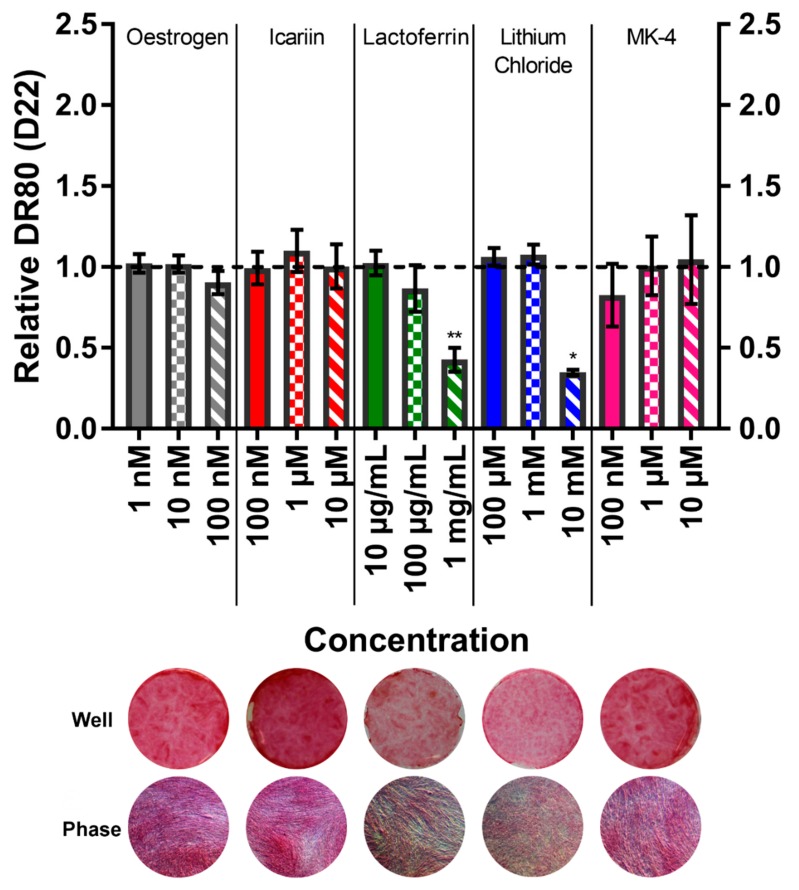
(**Top**) The effect of each compound and concentration on the collagen deposition at day 22, normalised to their respective vehicle control (horizontal dashed line). Lactoferrin (1 mg/mL) and lithium chloride (10 mM) significantly diminished the collagen deposition (p < 0.01 & p < 0.05, respectively). No other condition significantly deviated from the vehicle control. (n = 8). (**Bottom**) Representative whole well images (diameter ~15 mm) and high magnification (40×) phase contrast images (diameter 200 μm) of DR80 staining of each compound at the highest concentration applied.

The calcium deposition by ARS staining (Figure 6) and collagen deposition by DR80 staining (Figure 7) were quantified on day 22 to assess the mineralised matrix production. The total ARS and total DR80 per well for the vehicle controls were 421.8 ± 64.00 µg and 42.62 ± 5.98 µg, respectively. The treatment with lactoferrin at 1 mg/mL and lithium chloride at 10 mM both caused significantly less calcium to be deposited. Interestingly, phase contrast imaging reveals that although lactoferrin causes fewer and smaller mineral nodules to be formed, they appear normal in morphology. However, with lithium chloride, ARS staining is present throughout the well, but it appears much lighter/pinker macroscopically. At high magnifications, this is revealed to be due to calcium not depositing as nodules but as highly disordered, tangled, ‘spaghetti-like’ structures. This is consistent with collagen deposition, where with lactoferrin a smaller quantity of normal collagen deposition is present, whereas with lithium chloride it appears speckled and broken up. The application of 10 µM MK-4 resulted in 50% more mineralisation (p < 0.05) with no effect on the collagen production, demonstrating an anabolic, osteogenic effect. All mineral and collagen staining images can be found in the Supplementary Materials. 

## 4. Discussion

In this study, five reportedly osteogenic compounds were compared under controlled conditions to allow for a direct comparison and to determine their relative osteogenic effects on hES-MPs, a commercially available mesenchymal progenitor cell line that is a demonstrated model of adult MSCs. Our initial hypothesis was that these compounds would stimulate a bone-like matrix formation *in vitro* in these mesenchymal progenitors and have potential for use in bone tissue engineering, as well as for bone regeneration *in vivo.*

Of the five compounds investigated here, only 10 µM menaquinone-4 (MK-4) resulted in a greater mineralised matrix deposition in hES-MPs. This agrees with recent similar studies that showed the same concentration of MK-4 elevated ALP activity and mineralisation in human amniotic fluid MSCs in a monolayer culture and in 3D scaffolds seeded with dental pulp stem cells [42,43]. MK-4 is the most abundant isoform of menaquinone (vitamin K2) found in humans. Menaquinones have been shown to have both pro-osteoblastic and anti-osteoclastogenic attributes, and have received considerable attention for their positive effects on bone health [44,45,46,47,48]. Due to MK-4 having the effect of improving the bone quality rather than only retaining the bone mineral density, it has been prescribed as a first line treatment for osteoporosis with vitamin D since 2011 [49], although this is not yet routine outside Japan. This work, in combination with other recent findings, demonstrates its potential as an anabolic therapeutic for bone and as a useful molecule in bone tissue engineering [50,51].

The proposed mechanism of action of MK-4 is through targeting the steroid and xenobiotic sensing nuclear receptor (SXR)/pregnane X receptor (PXR) in order to stimulate osteoblast differentiation and facilitate bone formation [52,53,54], and the γ-carboxylation of non-collagenous bone proteins, specifically osteocalcin [55]. These processes promote differentiation and facilitate mineralisation; hence the increase in ALP activity and calcium deposition observed here and in other studies. Future work should investigate whether this mechanism of action is the reason for the increased mineralised matrix formation observed here. In this study, MK-4 only promoted mineralisation, with no observed increase in collagen deposition. However, if implemented for clinical use, this would not necessarily risk the same hypermineralisation and atypical fracture side effect observed with bisphosphonates [56], as tissue-engineered bone is often undermineralised in comparison to native bone, requiring extended time periods spanning many months of culture to achieve comparable levels of mineralisation [2,57,58]. 

Nevertheless, this means that the findings for the four other compounds disagree with some of the previous literature. Potential reasons for these discrepancies will be explored here. The role of oestrogen in protecting the skeletal bone composition is well known. 17β-estradiol is the most abundant of the oestrogens and the primary type produced by the ovaries. Its decline post-menopause is key in the pathogenesis of post-menopausal osteoporosis, primarily due to its effects on osteoclast-mediated bone resorption. In men, oestrogen is synthesised from testosterone via the enzyme ‘aromatase’. Oestrogen can act directly on monocytes, reducing their ability to become osteoclasts by disrupting the JNK pathway and upregulating apoptosis, as well as indirectly by reducing the RANKL:OPG ratio in osteoblast-lineage cells [59]. Whether oestrogen also has an anabolic effect on osteoblasts has been queried for over 20 years; however, *in vitro* studies have not definitively demonstrated that oestrogen is capable of increasing bone formation. There is evidence that it is pro-osteoblastic, for example by being anti-apoptotic [60] and by inducing ALP transcription [61]. However, anabolic effects have been predominantly reported in rodent and osteosarcoma cells [5,30], both of which are a poor representation of normal human MSCs/osteoblasts. One study did see anabolic effects in human MSCs [31], although it only used a single donor, indicating that the anabolic effects of oestrogen may be specific to the cell type or the donor, limiting its potential for use as an osteoblast-acting therapeutic. Interestingly, a recent study assessed 17β-estradiol synthesis from testosterone via aromatase by multiple donors of human MSCs. Up to 1 nM 17β-estradiol was synthesised by MSCs when cultured under osteogenic conditions with testosterone, comparable to the concentrations that were used here. No effect on the ALP activity was observed; however, osteogenic conditions with testosterone and the subsequent oestrogen production caused a significant increase in mineralisation. However, this effect was not compared to the direct addition of 17β-estradiol without testosterone, leaving it unclear whether the androgen or the oestrogen was the dominant cause of this increased mineralisation [62].

Icariin is the main flavonoid glycoside in the Epimedium herb and a phytoestrogen. Therefore, the similar finding to oestrogen that it had no detectable effect on hES-MPs other than a small increase in proliferation in the second and fourth week at 1 µM is not surprising. Interest in this molecule stems from its use in Chinese herbal medicine, and reports of its efficacy in promoting osteogenic differentiation and bone formation have been increasing in recent years. As with oestrogen, many of the studies demonstrating anabolic effects are performed using rodent cells [32,34], although it has also been reported to have an effect in a human foetal osteoblast cell line [63]. Interestingly, it has been shown *in vivo* that icariin is metabolised to icariside II and then icaritin prior to absorption and that these metabolites have significantly greater anabolic effects on osteoblasts *in vitro* than icariin does [33]. As icariin metabolism cannot occur in these single cell type cultures, this could be a reason why an effect was not detected. 

Lactoferrin is an iron binding protein and member of the transferrin family found in high concentrations in milk and colostrum. It is thought to promote osteoblast proliferation and differentiation by interaction with the LRP1 receptor and activation of p42/44 MAPK signalling [64]. In contrast to previous studies [36,37,38], lactoferrin was here found to have no effect on osteogenic differentiation at concentrations of 100 µg/mL and below, whilst 1000 µg/mL significantly reduced the proliferation, ALP activity, and mineralised matrix deposition. Where mineralised nodules did form at 1000 µg/mL, they appeared morphologically normal but were very small. Whilst bovine-derived lactoferrin was used in this study with human-origin cells, it has previously been shown that bovine, human, and recombinant forms have comparable effects; therefore, this is unlikely to be the reason for not detecting any anabolic effect [36]. Previous reports of an increasing ALP activity in response to lactoferrin have shown an immediate increase after only 24 hours of exposure in normal human osteoblasts, MC3T3-E1, and primary murine osteoblasts [38,65], but the effects were not examined at later time points. A continuous exposure for 35 days at concentrations of up to 1000 µg/mL showed a dose-dependent increase in mineralisation in the two murine cell types [65], although minimal mineralisation was observed in the absence of lactoferrin despite the use of 10 mM βGP. This high concentration of βGP has since been associated with the deposition of non-osteoblast mediated mineral due to a spontaneous precipitation of calcium phosphate [66]; therefore, it is possible that lactoferrin was influencing this mechanism rather than osteoblastic mineralisation. An increased matrix deposition has also been observed in the osteosarcoma cell line MG-63 [67,68], but these cancer cells are a poor representation of normal human MSCs/osteoblasts. 

Since the 1970s, lithium salts, specifically lithium chloride (LiCl), have been used as the gold standard treatment of bipolar disorders. It also has been found that patients treated with lithium for psychiatric disorders develop a higher bone mineral density [69], and the risk of bone fracture is reduced [70]. The precise mechanism by which lithium salts promote bone formation is yet to be elucidated, but one possibility is its inhibition of glycogen synthase kinase-3β and the consequent activation of canonical Wnt signalling [71]. In mesenchymal progenitors, this signalling inhibits adipogenesis and promotes osteoblastogenesis by regulating the expression of transcription factors such as RUNX2. However, another possible reason for the observed increase in BMD and reduced fracture risk in osteoporotic patients taking lithium is an increase in mechanosensitivity. Lithium chloride can lengthen the primary cilia, a mechanosensor present on most mammalian cells that bends in response to fluid flow [72], increasing its ability to transduce mechanical signals to cellular responses. Whilst a significant increase in the ALP activity was observed when 10 mM LiCl was applied in this study, the mineralised matrix deposition and cell number were significantly lower than the vehicle control. This effect is not surprising as serum concentrations this high are toxic, with the therapeutic serum concentration typically being 0.8–1.2 mM [39]. Furthermore, comparable LiCl concentrations have been shown to induce cell cycle G2/M phase arrest, potentially revealing why the metabolic activity and total DNA content were lower at 10 mM [73]. Although a mineralised matrix was deposited under 10 mM conditions, the collagen network and mineral were abnormal. Whilst collagen is normally produced in a ‘swirl’ pattern, here it appeared to be more speckled and discontinuous. This resulted in ‘spaghetti-like’ mineral patterns, rather than a typical nodule formation. 

The lack of response observed at lower concentrations may have been due to the influence of dexamethasone in the culture media, as this glucocorticoid (used to induce osteogenic differentiation) also acts via canonical Wnt signalling and the increase of RUNX2; therefore, its effects could have been masked [1]. Alternatively, the static culture employed in this study may have meant that the increase in mechanosensitivity that occurs after lithium chloride was not exploited and that, therefore, no increase in the mineralised matrix deposition was observed. 

In summary, of the five investigated compounds, only menaquinone-4 had an anabolic effect on hES-MPs. These findings are in close agreement with the recent literature that also investigated its effects on highly relevant osteoblast-lineage cells and emphasises its potential as an anabolic compound that can be utilised in bone tissue engineering. The lack of a detectable effect of oestrogen and icariin and the negative effects of high concentrations of lactoferrin and lithium chloride, when compared to the existing literature, highlights the variability caused by differences in experimental design and the relevance of the cell type that is used. 

## Figures and Tables

**Table 1:** The different compounds and concentrations investigated.

Compound	Low	Medium	High	Citation
Oestrogen	1 nM	10 nM	100 nM	[5,30,31]
Icariin	100 nM	1 µM	10 µM	[32,33,34,35]
Lactoferrin	10 µg/mL (~111 nM)	100 µg/mL (~1.11 µM)	1 mg/mL (~11.11 µM)	[36,37,38]
Lithium Chloride	100 µM	1 mM	10 mM	[39,40,41]
Menaquinone-4	100 nM	1 µM	10 µM	[42,43]

## Supplementary Materials

The following are available online at https://www.mdpi.com/2306-5354/7/1/12/s1, Figure S1: Mineral Pictures, Figure S2: Collagen Pictures, Table S1: Well plate layout for mineral and collagen studies.

## References

[1] Langenbach F., Handschel J. (2013). Effects of dexamethasone, ascorbic acid and beta-glycerophosphate on the osteogenic differentiation of stem cells in vitro. Stem Cell Res. Ther..

[2] Iordachescu A., Williams R.L., Hulley P.A., Grover L.M. (2019). Organotypic Culture of Bone-Like Structures Using Composite Ceramic-Fibrin Scaffolds. Curr. Protoc. Stem Cell Biol..

[3] James A.W., LaChaud G., Shen J., Asatrian G., Nguyen V., Zhang X., Ting K. (2016). and Soo, C. A Review of the Clinical Side Effects of Bone Morphogenetic Protein-2. Tissue Eng. Part B Rev..

[4] Diefenderfer D.L., Osyczka A.M., Reilly G.C., Leboy P.S. (2003). BMP Responsiveness in Human Mesenchymal Stem Cells. Connect. Tissue Res..

[5] Rao L.G., Liu L.J.F., Murray T.M., McDermott E., Zhang X. (2003). Estrogen added intermittently, but not continuously, stimulates differentiation and bone formation in SaOS-2 cells. Biol. Pharm. Bull..

[6] Robinson J.A., Harris S.A., Riggs B.L., Spelsberg T.C. (1997). Estrogen regulation of human osteoblastic cell proliferation and differentiation. Endocrinology.

[7] Chen F.P., Lee N., Wang K.C., Soong Y.K., Huang K.E. (2002). Effect of estrogen and 1α, 25 (OH) 2-vitamin D3 on the activity and growth of human primary osteoblast-like cells in vitro. Fertil. Steril..

[8] Patlas N., Zadik Y., Yaffe P., Patlas M., Schwartz Z., Ornoy A. (2005). The response to sex steroid hormones and vitamin D of cultured osteoblasts derived from ovariectomized mice with and without 17β-estradiol pretreatment. Odontology.

[9] Park J.-B. (2012). Effects of low doses of estrone on the proliferation, differentiation and mineralization of osteoprecursor cells. Exp. Ther. Med..

[10] Clover J., Gowen M. (1994). Are MG-63 and HOS TE85 human osteosarcoma cell lines representative models of the osteoblastic phenotype?. Bone.

[11] Czekanska E.M., Stoddart M.J., Richards R.G., Hayes J.S. (2012). In search of an osteoblast cell model for in vitro research. Eur. Cell Mater..

[12] De Peppo G.M., Svensson S., Lennerås M., Synnergren J., Stenberg J., Strehl R., Hyllner J., Thomsen P., Karlsson C. (2010). Human Embryonic Mesodermal Progenitors Highly Resemble Human Mesenchymal Stem Cells and Display High Potential for Tissue Engineering Applications. Tissue Eng. Part A.

[13] Duffy C.R.E., Zhang R., How S.E., Lilienkampf A., De Sousa P.A., Bradley M. (2014). Long term mesenchymal stem cell culture on a defined synthetic substrate with enzyme free passaging. Biomaterials.

[14] De Peppo G.M., Sjovall P., Lennerås M., Strehl R., Hyllner J., Thomsen P., Karlsson C. (2010). Osteogenic Potential of Human Mesenchymal Stem Cells and Human Embryonic Stem Cell-Derived Mesodermal Progenitors: A Tissue Engineering Perspective. Tissue Eng. Part A.

[15] Puwanun S. (2014). Developing a tissue engineering strategy for cleft palate repair. Ph.D. Thesis.

[16] Owen R., Sherborne C., Paterson T., Green N.H., Reilly G.C., Claeyssens F. (2016). Emulsion templated scaffolds with tunable mechanical properties for bone tissue engineering. J. Mech. Behav. Biomed. Mater..

[17] Owen R., Sherborne C., Reilly G.C., Claeyssens F. (2015). Data for the analysis of PolyHIPE scaffolds with tunable mechanical properties for bone tissue engineering. Data Brief.

[18] Paterson T.E., Gigliobianco G., Sherborne C., Green N.H., Dugan J.M., MacNeil S., Reilly G.C., Claeyssens F. (2018). Porous microspheres support mesenchymal progenitor cell ingrowth and stimulate angiogenesis. APL Bioeng..

[19] Tetteh G., Khan A., Delaine-Smith R., Reilly G., Rehman I. (2014). Electrospun polyurethane/hydroxyapatite bioactive Scaffolds for bone tissue engineering: The role of solvent and hydroxyapatite particles. J. Mech. Behav. Biomed. Mater..

[20] Qasim S.B., Delaine-Smith R.M., Fey T., Rawlinson A., Rehman I.U. (2015). Freeze gelated porous membranes for periodontal tissue regeneration. Acta Biomater..

[21] Qasim S.B., Najeeb S., Delaine-Smith R.M., Rawlinson A., Rehman I.U. (2017). Potential of electrospun chitosan fibers as a surface layer in functionally graded GTR membrane for periodontal regeneration. Dent. Mater..

[22] Puwanun S., Bye F.J., Ireland M.M., MacNeil S., Reilly G.C., Green N.H. (2016). Production and Characterization of a Novel, Electrospun, Tri-Layer Polycaprolactone Membrane for the Segregated Co-Culture of Bone and Soft Tissue. Polymers.

[23] Bye F.J., Bissoli J., Black L., Bullock A.J., Puwanun S., Moharamzadeh K., Reilly G.C., Ryan A.J., MacNeil S. (2013). Development of bilayer and trilayer nanofibrous/microfibrous scaffolds for regenerative medicine. Biomater. Sci..

[24] Bhaskar B., Owen R., Bahmaee H., Rao P.S., Reilly G.C. (2018). Design and Assessment of a Dynamic Perfusion Bioreactor for Large Bone Tissue Engineering Scaffolds. Appl. Biochem. Biotechnol..

[25] Viswanathan P., Ondeck M.G., Chirasatitsin S., Ngamkham K., Reilly G.C., Engler A.J., Battaglia G. (2015). 3D surface topology guides stem cell adhesion and differentiation. Biomaterials.

[26] Duffy C.R.E., Zhang R., How S.-E., Lilienkampf A., Tourniaire G., Hu W., West C.C., De Sousa P., Bradley M. (2014). A high-throughput polymer microarray approach for identifying defined substrates for mesenchymal stem cells. Biomater. Sci..

[27] Antonini L.M., Kothe V., Reilly G.C., Owen R., Marcuzzo J.S., Malfatti C.D.F. (2017). Effect of Ti6Al4V surface morphology on the osteogenic differentiation of human embryonic stem cells. J. Mater. Res..

[28] Delaine-Smith R.M., MacNeil S., Reilly G.C. (2012). Matrix production and collagen structure are enhanced in two types of osteogenic progenitor cells by a simple fluid shear stress stimulus. Eur. Cell Mater..

[29] Dana S., Bettina H., Matthias S., Andreas L., Seiler A.E. (2016). Osteogenic Differentiation of Human Embryonic Stem Cell-Derived Mesenchymal Progenitor Cells as a Model for Assessing Developmental Bone Toxicity In Vitro. Appl. In Vitro Toxicol..

[30] Brennan M., Haugh M., O’Brien F., McNamara L. (2014). Estrogen Withdrawal from Osteoblasts and Osteocytes Causes Increased Mineralization and Apoptosis. Horm. Metab. Res..

[31] Hong L., Colpan A., Peptan I.A. (2006). Modulations of 17-β Estradiol on Osteogenic and Adipogenic Differentiations of Human Mesenchymal Stem Cells. Tissue Eng..

[32] Chen K., Ge B., Ma H., Liu X., Bai M., Wang Y. (2005). Icariin, a flavonoid from the herb Epimedium enhances the osteogenic differentiation of rat primary bone marrow stromal cells. Pharm. Int. J. Pharm. Sci..

[33] Huang J., Yuan L., Wang X., Zhang T.L., Wang K. (2007). Icaritin and its glycosides enhance osteoblastic, but suppress osteoclastic, differentiation and activity in vitro. Life Sci..

[34] Ma H.P., Ming L.G., Ge B.F., Zhai Y.K., Song P., Xian C.J., Chen K.M. (2011). Icariin is more potent than genistein in promoting osteoblast differentiation and mineralization in vitro. J. Cell. Biochem..

[35] Zhao J., Ohba S., Shinkai M., Chung U.-I., Nagamune T. (2008). Icariin induces osteogenic differentiation in vitro in a BMP- and Runx2-dependent manner. Biochem. Biophys. Res. Commun..

[36] Cornish J., Callon K.E., Naot D., Palmano K.P., Banovic T., Bava U., Watson M., Lin J.-M., Tong P.C., Chen Q. (2004). Lactoferrin Is a Potent Regulator of Bone Cell Activity and Increases Bone Formation in Vivo. Endocrinology.

[37] Grey A., Banovic T., Zhu Q., Watson M., Callon K., Palmano K., Ross J., Naot D., Reid I.R., Cornish J. (2004). The Low-Density Lipoprotein Receptor-Related Protein 1 Is a Mitogenic Receptor for Lactoferrin in Osteoblastic Cells. Mol. Endocrinol..

[38] Li Q., Zhao J., Hu W., Wang J., Yu T., Dai Y., Li N. (2017). Effects of Recombinant Human Lactoferrin on Osteoblast Growth and Bone Status in Piglets. Anim. Biotechnol..

[39] Aral H., Vecchio-Sadus A. (2008). Toxicity of lithium to humans and the environment—A literature review. Ecotoxicol. Environ. Saf..

[40] Tang L., Chen Y., Pei F., Zhang H. (2015). Lithium Chloride Modulates Adipogenesis and Osteogenesis of Human Bone Marrow-Derived Mesenchymal Stem Cells. Cell. Physiol. Biochem..

[41] Yu Z., Fan L., Li J., Ge Z., Dang X., Wang K. (2015). Lithium chloride attenuates the abnormal osteogenic/adipogenic differentiation of bone marrow-derived mesenchymal stem cells obtained from rats with steroid-related osteonecrosis by activating the β-catenin pathway. Int. J. Mol. Med..

[42] Rasouli-Ghahroudi A.A., Akbari S., Najafi-Alishah M., Bohloli M. (2017). The Effect of Vitamin K2 on Osteogenic Differentiation of Dental Pulp Stem Cells: An In Vitro Study. Regen. Reconstr. Restor..

[43] Mandatori D., Penolazzi L., Pipino C., Di Tomo P., Di Silvestre S., Di Pietro N., Trevisani S., Angelozzi M., Ucci M., Piva R. (2018). Menaquinone-4 enhances osteogenic potential of human amniotic fluid mesenchymal stem cells cultured in 2D and 3D dynamic culture systems. J. Tissue Eng. Regen. Med..

[44] Puwanun S., Delaine-Smith R.M., Colley H., Yates J.M., MacNeil S., Reilly G.C. (2018). A simple rocker-induced mechanical stimulus upregulates mineralization by human osteoprogenitor cells in fibrous scaffolds. J. Tissue Eng. Regen. Med..

[45] Shiraki M., Shiraki Y., Aoki C., Miura M. (2000). Vitamin K2 (menatetrenone) effectively prevents fractures and sustains lumbar bone mineral density in osteoporosis. J. Bone Miner. Res..

[46] Ishida Y., Kawai S. (2004). Comparative efficacy of hormone replacement therapy, etidronate, calcitonin, alfacalcidol, and vitamin K in postmenopausal women with osteoporosis: The Yamaguchi Osteoporosis Prevention Study. Am. J. Med..

[47] Koitaya N., Sekiguchi M., Tousen Y., Nishide Y., Morita A., Yamauchi J., Gando Y., Miyachi M., Aoki M., Komatsu M. (2014). Low-dose vitamin K 2 (MK-4) supplementation for 12 months improves bone metabolism and prevents forearm bone loss in postmenopausal Japanese women. J. Bone Miner. Metab..

[48] Huang Z.B., Wan S.L., Lu Y.J., Ning L., Liu C., Fan S.W. (2015). Does vitamin K2 play a role in the prevention and treatment of osteoporosis for postmenopausal women: a meta-analysis of randomized controlled trials. Osteoporos. Int..

[49] Orimo H., Nakamura T., Hosoi T., Iki M., Uenishi K., Endo N., Ohta H., Shiraki M., Sugimoto T., Suzuki T. (2012). Japanese 2011 guidelines for prevention and treatment of osteoporosis—Executive summary. Arch. Osteoporos..

[50] Weng S.J., Xie Z.J., Wu Z.Y., Yan D.Y., Tang J.H., Shen Z.J., Li H., Bai B.L., Boodhun V., Dong X.D.E. (2019). Effects of combined menaquinone-4 and PTH1-34 treatment on osetogenesis and angiogenesis in calvarial defect in osteopenic rats. Endocrine.

[51] Li H., Zhou Q., Bai B.L., Weng S.J., Wu Z.Y., Xie Z.J., Feng Z.H., Cheng L., Boodhun V., Yang L. (2018). Effects of combined human parathyroid hormone (1–34) and menaquinone-4 treatment on the interface of hydroxyapatite-coated titanium implants in the femur of osteoporotic rats. J. Bone Miner. Metab..

[52] Sasaki N., Kusano E., Takahashi H., Ando Y., Yano K., Tsuda E., Asano Y. (2005). Vitamin K2 inhibits glucocorticoid-induced bone loss partly by preventing the reduction of osteoprotegerin (OPG). J. Bone Miner. Metab..

[53] Iwamoto J., Seki A., Sato Y., Matsumoto H., Tadeda T., Yeh J.K. (2010). Vitamin K2 Promotes Bone Healing in a Rat Femoral Osteotomy Model with or without Glucocorticoid Treatment. Calcif. Tissue Int..

[54] Tabb M.M., Sun A., Zhou C., Grün F., Errandi J., Romero K., Pham H., Inoue S., Mallick S., Lin M. (2003). Vitamin K2 Regulation of Bone Homeostasis Is Mediated by the Steroid and Xenobiotic Receptor SXR. J. Biol. Chem..

[55] Frandsen N.E., Gordeladze J.O. (2017). Vitamin K2-Vital for Health and Wellbeing.

[56] Lloyd A.A., Gludovatz B., Riedel C., Luengo E.A., Saiyed R., Marty E., Lorich D.G., Lane J.M., Ritchie R.O., Busse B. (2017). Atypical fracture with long-term bisphosphonate therapy is associated with altered cortical composition and reduced fracture resistance. Proc. Natl. Acad. Sci. USA.

[57] Iordachescu A., Amin H.D., Rankin S.M., Williams R.L., Yapp C., Bannerman A., Pacureanu A., Addison O., Hulley P.A., Grover L.M. (2018). Organotypic Bone Culture: An In Vitro Model for the Development of Mature Bone Containing an Osteocyte Network. Adv. Biosyst..

[58] Follet H., Boivin G., Rumelhart C., Meunier P. (2004). The degree of mineralization is a determinant of bone strength: a study on human calcanei. Bone.

[59] Owen R., Reilly G.C. (2018). In vitro Models of Bone Remodelling and Associated Disorders. Front Bioeng. Biotechnol..

[60] Kousteni S. (2001). Nongenotropic, Sex-Nonspecific Signaling through the Estrogen or Androgen Receptors Dissociation from Transcriptional Activity. Cell.

[61] Krum S.A., Miranda-Carboni G.A., Lupien M., Eeckhoute J., Carroll J.S., Brown M. (2008). Unique ERα cistromes control cell type-specific gene regulation. Mol. Endocrinol..

[62] Glenske K., Schuler G., Arnhold S., Elashry M.I., Wagner A.-S., Barbeck M., Neumann E., Müller-Ladner U., Schnettler R., Wenisch S. (2019). Effects of testosterone and 17β-estradiol on osteogenic and adipogenic differentiation capacity of human bone-derived mesenchymal stromal cells of postmenopausal women. Bone Rep..

[63] Liang W., Lin M., Li X., Li C., Gao B., Gan H., Yang Z., Lin X., Liao L., Yang M. (2012). Icariin promotes bone formation via the BMP-2/Smad4 signal transduction pathway in the hFOB 1.19 human osteoblastic cell line. Int. J. Mol. Med..

[64] Naot D., Grey A., Reid I.R., Cornish J. (2005). Lactoferrin — A Novel Bone Growth Factor. Clin. Med. Res..

[65] Zhang W., Guo H., Jing H., Li Y., Wang X., Zhang H., Jiang L., Ren F. (2014). Lactoferrin Stimulates Osteoblast Differentiation Through PKA and p38 Pathways Independent of Lactoferrin’s Receptor LRP1. J. Bone Miner. Res..

[66] Schäck L.M., Noack S., Winkler R., Wißmann G., Behrens P., Wellmann M., Jagodzinski M., Krettek C., Hoffmann A. (2013). The Phosphate Source Influences Gene Expression and Quality of Mineralization during In Vitro Osteogenic Differentiation of Human Mesenchymal Stem Cells. PLoS ONE.

[67] Takayama Y., Mizumachi K. (2008). Effect of Bovine Lactoferrin on Extracellular Matrix Calcification by Human Osteoblast-Like Cells. Biosci. Biotechnol. Biochem..

[68] Takayama Y., Mizumachi K. (2009). Effect of lactoferrin-embedded collagen membrane on osteogenic differentiation of human osteoblast-like cells. J. Biosci. Bioeng..

[69] Zamani A., Omrani G.R., Nasab M.M. (2009). Lithium’s effect on bone mineral density. Bone.

[70] Vestergaard P., Rejnmark L., Mosekilde L. (2005). Reduced Relative Risk of Fractures Among Users of Lithium. Calcif. Tissue Int..

[71] Clément-Lacroix P., Ai M., Morvan F., Roman-Roman S., Vayssière B., Belleville C., Estrera K., Warman M.L., Baron R., Rawadi G. (2005). Lrp5-independent activation of Wnt signaling by lithium chloride increases bone formation and bone mass in mice. Proc. Natl. Acad. Sci. USA.

[72] Wheatley D. (1996). Expression of primary cilia in mammalian cells. Cell Biol. Int..

[73] Yao R., Sun X., Xie Y., Liu L., Han D., Yao Y., Li H., Li Z., Xu K. (2018). Lithium chloride inhibits cell survival, overcomes drug resistance, and triggers apoptosis in multiple myeloma via activation of the Wnt/β-catenin pathway. Am. J. Transl. Res..

