# Supplementary material for: Comparison of the Anabolic Effects of Reported Osteogenic Compounds on Human Mesenchymal Progenitor-Derived Osteoblasts
Source: Bioengineering (Basel). 2020 Jan 21;7(1):12. doi: 10.3390/bioengineering7010012 (PMC7148480; doi:10.3390/bioengineering7010012)
Supplement: Supplementary file 1 [file bioengineering-07-00012-s001.zip › Supplementary Information.docx]

**Supplementary Information**

Whole well plate images of all wells assessed by alizarin red S (Fig. S1) and direct red 80 (Fig. S2) in figures 6 and 7. All plates were cultured in the same format (Table S1). Where two wells are missing for the vehicle column in repeat 1 of lithium chloride this is due to detachment of the cell monolayer.

*Table S1: Well plate layout for mineral and collagen studies*

| **Column Number (Left to Right)** | **Conditions** |
| --- | --- |
| 1 | OIM + VC |
| 2 | OIM + Low concentration |
| 3 | OIM + Medium concentration |
| 4 | OIM + High concentration |
