# Supplementary figures and images for: Comparison of the Anabolic Effects of Reported Osteogenic Compounds on Human Mesenchymal Progenitor-Derived Osteoblasts
Source: Bioengineering (Basel). 2020 Jan 21;7(1):12. doi: 10.3390/bioengineering7010012 (PMC7148480; doi:10.3390/bioengineering7010012)

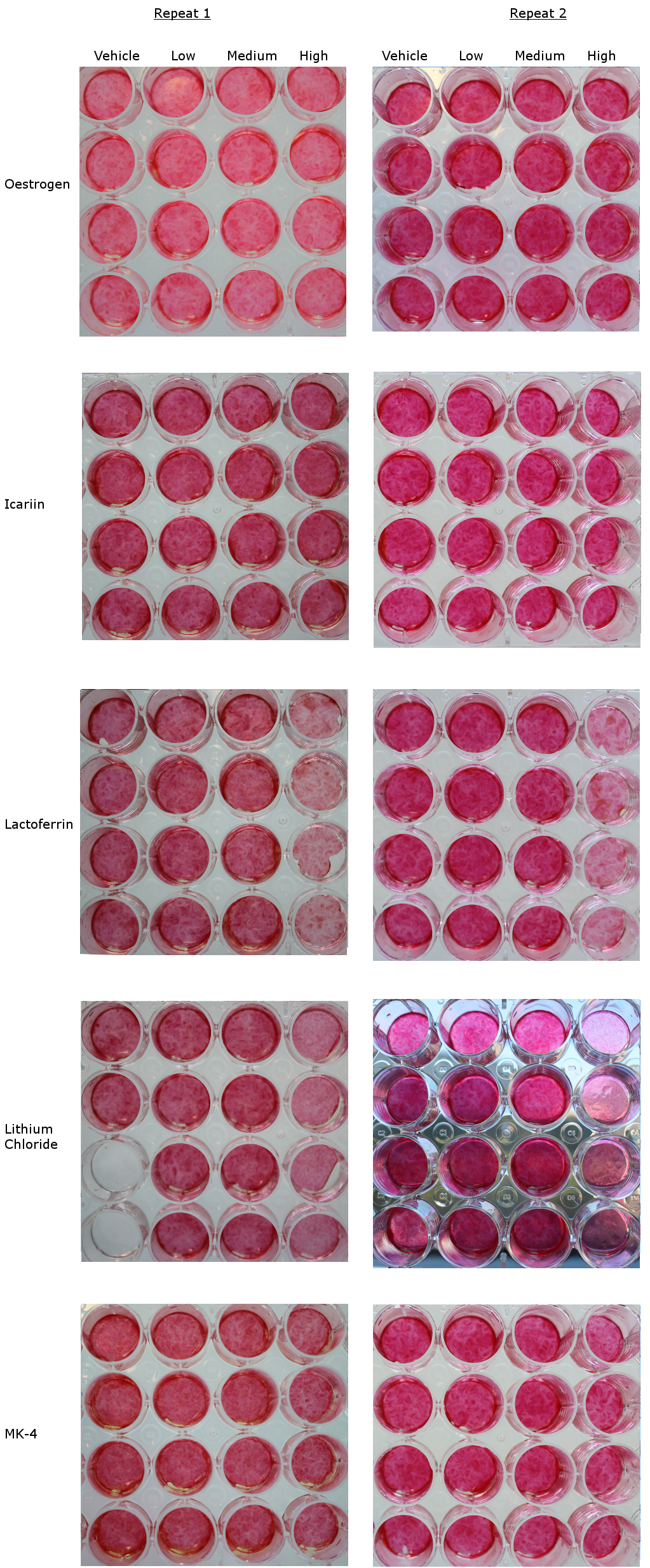

Supplement: Supplementary file 1 [file bioengineering-07-00012-s001.zip › Figure S2 - Collagen Pictures.tiff]

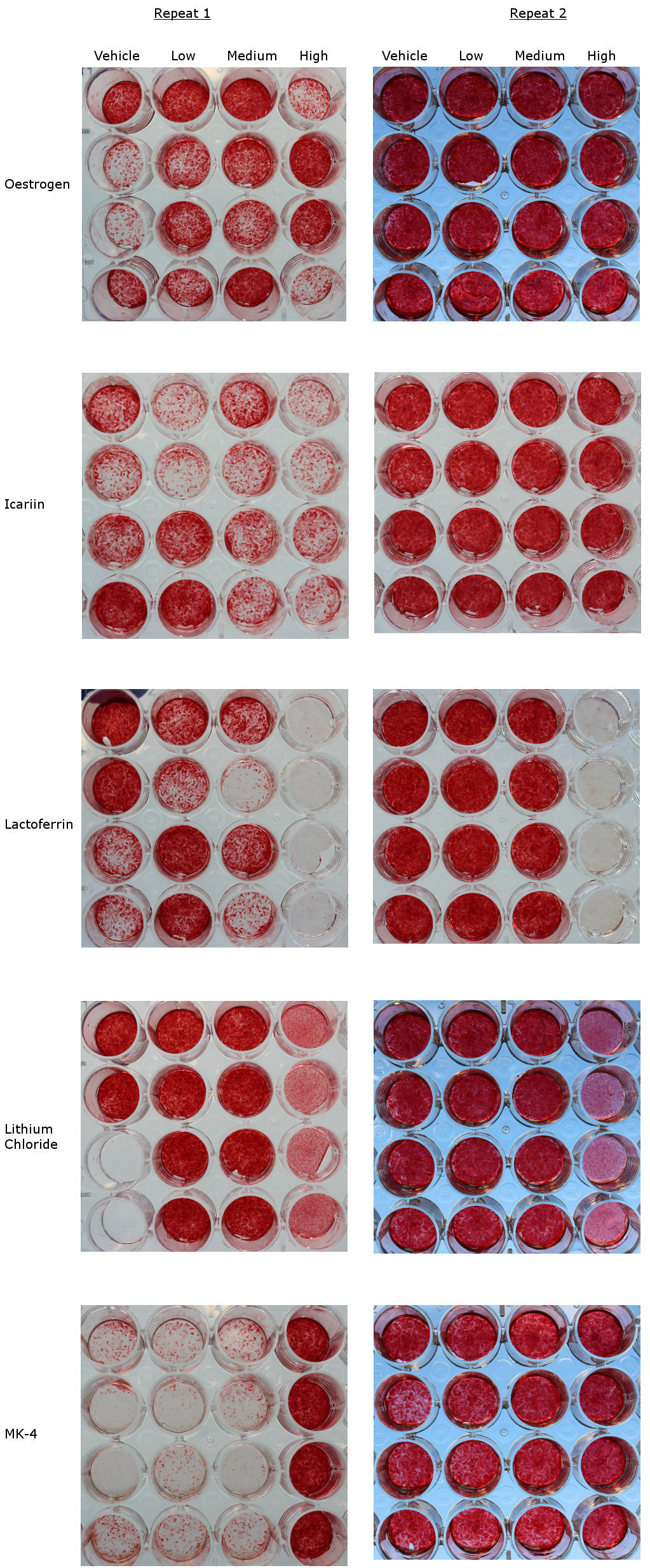

Supplement: Supplementary file 1 [file bioengineering-07-00012-s001.zip › Figure S1 - Mineral Pictures.tiff]
